# A Decision Support System for Water Optimization in Anti-Frost Techniques by Sprinklers

**DOI:** 10.3390/s20247129

**Published:** 2020-12-12

**Authors:** Miguel A. Guillén-Navarro, Raquel Martínez-España, Andrés Bueno-Crespo, Juan Morales-García, Belén Ayuso, José M. Cecilia

**Affiliations:** 1 Computer Science Department, Universidad Católica de Murcia (UCAM), 30107 Murcia, Spain; maguillen@ucam.edu (M.A.G.-N.); abueno@ucam.edu (A.B.-C.); jmorales8@alu.ucam.edu (J.M.-G.); bayuso@ucam.edu (B.A.); 2 Computer Engineering Department (DISCA), Universitat Politècnica de València (UPV), 46022 Valencia, Spain; jmcecilia@disca.upv.es

**Keywords:** multivariate LSTM based approach, IoT system, intelligent systems, precision agriculture

## Abstract

Precision agriculture is a growing sector that improves traditional agricultural processes through the use of new technologies. In southeast Spain, farmers are continuously fighting against harsh conditions caused by the effects of climate change. Among these problems, the great variability of temperatures (up to 20 ${}^{{}^{\circ}}$C in the same day) stands out. This causes the stone fruit trees to flower prematurely and the low winter temperatures freeze the flower causing the loss of the crop. Farmers use anti-freeze techniques to prevent crop loss and the most widely used techniques are those that use water irrigation as they are cheaper than other techniques. However, these techniques waste too much water and it is a scarce resource, especially in this area. In this article, we propose a novel intelligent Internet of Things (IoT) monitoring system to optimize the use of water in these anti-frost techniques while minimizing crop loss. The intelligent component of the IoT system is designed using an approach based on a multivariate Long Short-Term Memory (LSTM) model, designed to predict low temperatures. We compare the proposed approach of multivariate model with the univariate counterpart version to figure out which model obtains better accuracy to predict low temperatures. An accurate prediction of low temperatures would translate into significant water savings, as anti-frost techniques would not be activated without being necessary. Our experimental results show that the proposed multivariate LSTM approach improves the univariate counterpart version, obtaining an average quadratic error no greater than 0.65 ${}^{{}^{\circ}}$C and a coefficient of determination R${}^{2}$ greater than 0.97. The proposed system has been deployed and is currently operating in a real environment obtained satisfactory performance.

## 1. Introduction

Agriculture is increasingly applying new technologies to increase its market opportunities and improve the use of available resources. Without a doubt, the most necessary natural resource in agriculture is water. However, water is an increasingly scarce resource and must be used rationally in this climate change era [1]. Precision agriculture (PA) is a discipline that offers a set of tools and techniques that enable farmers to increase their production and the quality of their production, while reducing costs and the resources used [2]. PA integrates a set of approaches to manage and help make decisions by reducing the uncertainty caused by the variability of the agricultural field. One of the uncertainties that create the most problems in agriculture is the sudden changes in the weather [3]. Among these abrupt changes are the variability of temperatures or torrential rainfall, causing great economic losses to both farmers and the sectors that depend on them [4]. In particular, this situation is particularly damaging in the southeast of the Mediterranean area, where crops can suffer temperature variations of up to 20 degrees centigrade (°C) within the same day. These ups and downs lead to spring temperatures in late autumn and early winter and low temperatures in early spring. These changes in the weather cause the fruit trees to bloom early and with the low temperatures in early spring the flowers freeze. To avoid crop loss, farmers apply different anti-frost techniques, depending on the type of crop [5]. Anti-frost techniques can be classified as active or passive. Active techniques are temporary and are energy or labor intensive, or both. Passive techniques are related to biological and ecological techniques, including practices carried out before a night of frost to reduce damage. Passive techniques are based on carrying out soil analysis, choosing suitable seeds in the plantations, genetic modifications in the plants, etc. The active techniques use micro-spraying, flood irrigation, stoves, covers, fans, and smoke and fog [6]. This study focuses on active anti-frost techniques, specifically those that use water as an anti-frost agent.

In addition to the meteorological problems, there are also water scarcity issues. Farmers in many areas are restricted in the amount of water available to irrigate their crops, so if they use disproportionate amounts of water for anti-frost techniques, they could save their crops but have no water available to harvest them. Although other anti-frost techniques could be used, most of them require a high installation cost and the amortization is a high burden for the farmers. The techniques that use water are the cheapest in installations, hence it is one of the most used. Therefore, the main objective of this study is to find a model that helps to predict when the temperature in the next hour with an error not exceeding one Celsius degree. This model will be implemented within the intelligent component of an IoT system that will have actuators to activate or deactivate the anti-frost techniques and thus save the maximum amount of water available. However, frost prediction is not an easy task, as it depends on several external environmental factors that are very difficult to control, such as temperature, humidity, wind speed, etc. In addition, there are also factors such as the location of the plot that are involved in this process. General weather forecasting provides information on a large scale which, on many occasions, is not sufficient to predict frost at plot level. At least, not with the precision to determine when to apply anti-frost techniques. Therefore, the particular conditions of each region of the plot must be taken into account in order to obtain more accurate predictive models.

Indeed, IoT systems play a fundamental role to monitor the climatic conditions of the crops automatically in a given area within a plot. IoT alone is not enough to provide an effective solution to this problem. The integration of both Artificial Intelligence (AI) and IoT is mandatory to enable such innovation. AI-enabled IoT (AIoT) brings sensors, machines, cloud-edge computing, analytics, and people together to improve productivity and efficiency, which implies revenue growth and operational efficiency. However, AI techniques, and, particularly, Machine Learning (ML) models involve computationally intensive tasks that also require high-quality data. Moreover, there are several challenges which limit the AIoT implementation in rural areas. Among them, we may highlight technical issues such as bandwidth, connectivity, power supply, and scaling sensors but also environmental issues that complicate the developments, such as extreme weather conditions.

This paper introduces an AIoT system to predict the air temperature based on several input variables. The aim of this AIoT is for farmers to activate their anti-frost techniques based on the use of water only when necessary, thus achieving significant savings of this scarce resource. Thus, we have designed and implemented a model based on a multivariate deep learning model based on Long Short-Term Memory (LSTM) to be integrated into an IoT system deployed in several agricultural plots. In the design of the model, the different alternatives have been evaluated, trying to implement the AIoT system in any plot without the need for significant historical data. In addition, the capacity of the proposed LSTM model to obtain a more precise result depending on the number of climate variables used is also analyzed. Thus, once the model has been assessed and validated, its aim is to activate the actuators and alert the farmer with sufficient precision and time so as not to lose the harvest, taking into account water savings. The LSTM model proposed in this article, deployed on the IoT infrastructure, creates a decision support system that activates and deactivates anti-freeze sprinkler techniques.

In what follows, the main research objectives of this manuscript are addressed:

1. Saving water in anti-frost techniques: One of the main objectives is to design and deploy a hardware and software AIoT infrastructure to monitor and predict temperatures in order to activate the anti-frost techniques based on sprinkler irrigation only when they are strictly necessary, taking care with the use of such a precious good as water.
2. Designing an accurate intelligent component through LSTM models: To achieve the previous goal, a multivariate LSTM model is proposed and evaluated to provide temperature predictions accurately.
3. Data characterization: Analyze the seasonality and amount of historical data needed to establish the system in other environments.
4. Integration and deployment: Integrate the data prediction model together with the IoT system to do the job of activating actuators and saving water.

Each of these objectives is described, detailed and addressed in Section 3 of this manuscript. Thus, this study is organized as follows. First, a brief background is given on aspects related to deep learning and precision agriculture in Section 2. In Section 3, the description and architecture of the AIoT infrastructure is provided, and the prediction model used (LSTM) is explained in depth. It also describes the datasets and variables used to analyze the historical data and the configuration of the experiments carried out to create the prediction model. Then, Section 4 provides the results of an analysis of them and a discussion of the objectives achieved are presented. Finally, some conclusions and orientations for future work are explained.

## 2. Related Work

This section briefly summarizes the state of the art of precision agriculture techniques in the field of water management. It is well known that the development of intelligent environments for application to precision agriculture is widespread, and this is an appropriate field of action as described in [7]. A first interesting work is [8], where a model composed of an LSTM layer with another fully connected layer on top of it is developed to predict water table depth. They use a dropout method applied in the first LSTM layer. This model is evaluated in China with monthly data during 14 years of water diversion, evaporation, precipitation, and temperature. Moreover, it could help engineers and decision-makers to plan and manage groundwater resources in agricultural areas. The use of such a model in the field of hydrology is very novel and shows that there are areas where different models can be applied to a traditional neural network. The results obtained from the proposed model indicate a value of R^2^ of 0.86. Sahoo et al. [9] proposed a model which predicts the level of groundwater in an area of the United States. Since the demand for irrigation water influences the wells in the area from which the water is drawn, farmers can know the level of the groundwater before using this water. The results show a correlation value in the model greater than 0.8.

Another work that demonstrates to what extent Artificial Intelligence can help solve everyday situations but with an important economic impact on small farms is described in [10]. Coopersmith et al. model the process by which the soil becomes wet and dry. This can help farmers decide when is a suitable time to bring in heavy machinery. They have used only publicly-accessible information and classification trees, k-nearest-neighbors and boosted perceptron deliver statistical soil dryness estimates. The authors achieve an accuracy value of 94% with the proposed model. In terms of water management, there are several articles which propose evapotranspiration and evaporation methodologies. For instance, Patil and Deka show an accurate estimation of weekly evapotranspiration in arid regions of India using a method developed by them and based on the ELM model fed with temperature data for the weekly estimation of evapotranspiration for two weather stations [11]; Mohammadi et al. show that the dew point is a very significant element for the identification of meteorological phenomena (evapotranspiration, evaporation, and frost) [12]. They introduce a model for the prediction of the daily dew point temperature based on the LMA. Feng et al. show two scenarios where the estimation of daily evapotranspiration from temperature data collected at six weather stations was carried out [13]. Finally, in [14], a recurrent LSTM neural network for the forecast of the Cimandiri River level in Indonesia is presented, and this model achieves a relative error of less than 10%.

The authors of [15] present an approach to predict temperature and humidity using a two-level sequential decomposition structure. First, the meteorological data were decomposed into four components in series. Then, each of these series is introduced to a gated recurrent unit, and the output information from each of these units is combined to obtain the prediction results. The data used to test the proposed approach are collected using a IoT system. The results indicate a root mean square error (RMSE) greater than 2.4 degrees Celsius for temperature and greater than 13% for humidity prediction. In [16], an intelligent frost management system is created. The connection in real time is guaranteed by the use of a web that allows the interaction of the environmental system with the weather station and the ecological anti-freeze irrigation. The system uses a neural network and a Fuzzy Expert System—the former to optimally predict the temperature inside the greenhouses and the latter to control the activation of a water pump. Obtaining $R^{2}$ values for the temperature in the summer of 0.91 and in the winter of 0.95. The authors of [17] deploy the LSTM model for temperature prediction and propose the transductive LSTM (T-LSTM) by altering the cost function in the regression problem. With the T-LSTM model, they obtain a root mean square error greater than 1.5 degrees centigrade in the prediction of temperature.

To the best of our knowledge, although there are some articles that use LSTM to deal with agriculture issues, there is no study that addresses frost prediction through LSTM techniques. Specifically, the LSTM model proposed in this article does not require a great amount of historical data to obtain robust and reliable temperature predictions. This guarantees that it can be applied in different plots for short-term predictions.

## 3. AIoT System Proposed

As in other economic sectors, IoT technology has revolutionized the agricultural sector [18,19]. This is mainly due to the reduction in size, energy consumption, and cost of hardware components, which has democratized the use of these infrastructures in this sector. The IoT system proposed in this article monitors the climatic data of an agricultural plot and, based on the data and the intelligent system developed, the system acts by activating the anti-frost system and alerting the farmer to make the right decisions if necessary. The main objective of this system is to optimize the use of water in the anti-frost system, effectively predicting the air temperature by means of deep learning techniques included in the intelligent module.

**Figure 1:**
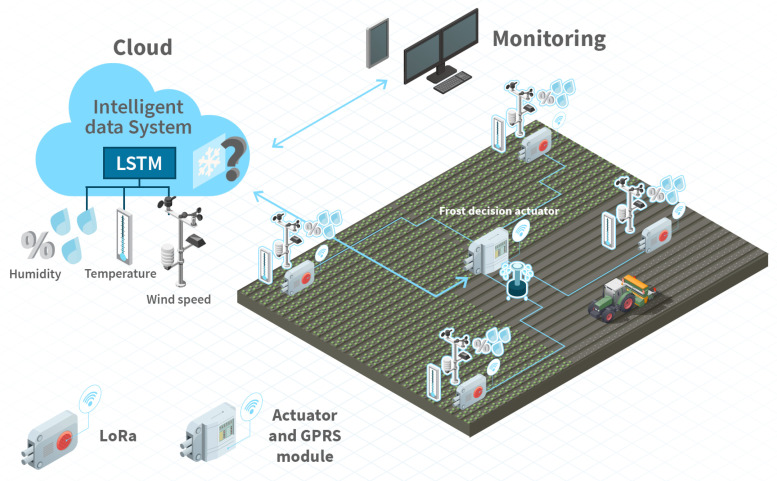
AIoT system architecture.

Figure 1 shows the AIoT infrastructure proposed in this article. The system consists of three different components: an infrastructure of agricultural sensors and actuators, an intelligent data processing system, and a monitoring component. The IoT infrastructure has three main sub-modules with three sensors connected to each of them (i.e., humidity, temperature, and wind speed). Each submodule communicates with the central module (actuator) using LoRa technology. LoRa enables long-distance communications, which allows the sensors to be dispersed in the agricultural field, without having to leave the local network. The actuator sends data to the cloud, where the proposed LSTM model will indicate the expected temperature in order to decide whether the actuator activates or deactivates the anti-frost technique, i.e., the sprinklers. In what follows, we introduce each of these components in detail.

### 3.1. Agricultural Sensors and Actuators Infrastructure

Firstly, an IoT infrastructure has been deployed in a real agricultural field, located in Cieza (Region of Murcia, Spain). This infrastructure is based on a wireless sensor network that collects (WSN) data on temperature, humidity, and wind speed. These data are sent via GPRS technology to the intelligent component of the system, to decide if the actuators should start remotely activating the anti-frost technique. One of the most relevant aspects and the one that has given more analysis to design the proposed IoT solution has been the choice of the communication technology between the different nodes.

#### 3.1.1. Analyzing the Wireless Communication Technologies

The wireless communication technology is one of the most important decisions in these scenarios. There are many IoT communication protocols which offer different characteristics for the efficient interconnection of IoT nodes [20]. However, the agriculture scenario requires long transmission distance, has freedom of use, low power consumption, minimal data transfer and is feasible to implement in sensors, actuators, and nodes outdoors. There are several data transmission standards which meet these characteristics such as SigFox (https://www.sigfox.com/), LoRa (https://lora-alliance.org/), or ZigBee (http://www.zigbee.org/). In our previous work, we tested ZigBee for connecting our IoT infrastructure since it is one of the most popular technologies in the field of agriculture [21,22]. Although its technical specifications indicate a communication range of 100 m, our initial tests did not show a communication range of more than 60 m [23].

Thus, a second scenario using the LoRa communication protocol was developed as a means to communicate with the IoT nodes. Among the advantages offered by this standard is the low power consumption and the great distance it reaches, in addition to being a free use protocol. The choice of using LoRa instead of Sigfox is based on the possibility of deploying our own network, since Sigfox imposes certain restrictions on its use. Therefore, the technology selected and implemented in the IoT solution for minimizing frost damage has been the LoRa communication protocol.

#### 3.1.2. IoT Sensor Hardware Description

The hardware architecture of the sensor network was previously explained in [22,24]. It is based on the 4H remote control system of the company Hidroconta (http://www.hidroconta.com/). The IoT node includes air temperature, humidity and wind speed sensors. These sensors have been calibrated by the company, so we have not had to perform any calibration phase. We briefly described them below:

- IoT nodes: The IoT architecture is composed of three nodes (a master and two slaves), where the sensors are connected to (see LoRa node in Figure 1). The master node is the gateway to send the data to the cloud through the GPRS connection. The connection of the slave nodes with the master node and the actuator is also done by LoRa technology. The other two nodes are connected to the master through LoRa. These nodes are autonomous since they have a 6-volt (V), 12-amp (A) per hour battery and a 12 V, 5-watt (W) solar panel. In addition, each IoT node has 96 KB of volatile data memory and a microcontroller with 256 KB of firmware storage. The former can be extended with an external non-volatile memory of 244 KB, which can store more than 20,000 registers. Therefore, each node stores information in its own memory besides sending the information to the gateway. Thus, if there is a communication failure, the information can be resent. Finally, the sensors and actuators are connected to the analog inputs available at the IoT node.
- Sensors and Actuators: The sensors and actuator components used in the proposed IoT solution are the following. The humidity sensor is COMET P3110E (https://www.cometsystem.com/) which also provides air temperature measurements. The temperature lies between −30 °C to 80 °C with an error of 0.6 °C and the humidity lies between 0 to 100% with an error of 3%. The temperature sensor is COMET 60P8610. The air temperature range lies between −20 °C to 60 °C with a resolution of 0.1 °C. The wind speed sensor is the PCE Instruments (https://www.pce-instruments.com/), model PCE-WS, whose measurement range is between 3 and 180 km/h with an error of km/h.

In this article, more variables than temperature have been included in order to carry out an in-depth analysis of the data and thus analyze the impact of having a multivariate model on the prediction of frost several hours in advance. Having two air temperature sensors (i.e., humidity sensor and air temperature) also provides the detection of outliers and/or temperature errors. Moreover, it also includes the possibility to detect the lowest temperature to activate the anti-frost technique. Actuator: The actuator is a valve that activates and deactivates the anti-freeze spray technique. It is a lach type solenoid which has three different modes of operation, resistance up to 80 °C, and 12 bar pressure.

### 3.2. IoT Intelligent Component

An IoT system needs an intelligent component to provide support on the farmer’s decision. The IoT intelligent component presented in this article includes a deep learning model based on Long Short-Term Memory neural networks (LSTM), specifically designed to work with the input provided by the IoT system developed. These input data are nonlinear times series where deep learning models have shown very good results [25]. The use of recurrent neural networks (RNN) with time series is a very powerful tool because it allows information to persist through a loop in the network diagram, allowing for remembering previous states and using this information to decide which will be the next stage to carry out. However, conventional RNNs usually have problems in their training due to the accumulation of errors that are generated, since it depends not only on the current error but also on past errors, and this makes them not very efficient for the memorization of dependencies in the long term sequences.

**Figure 2:**
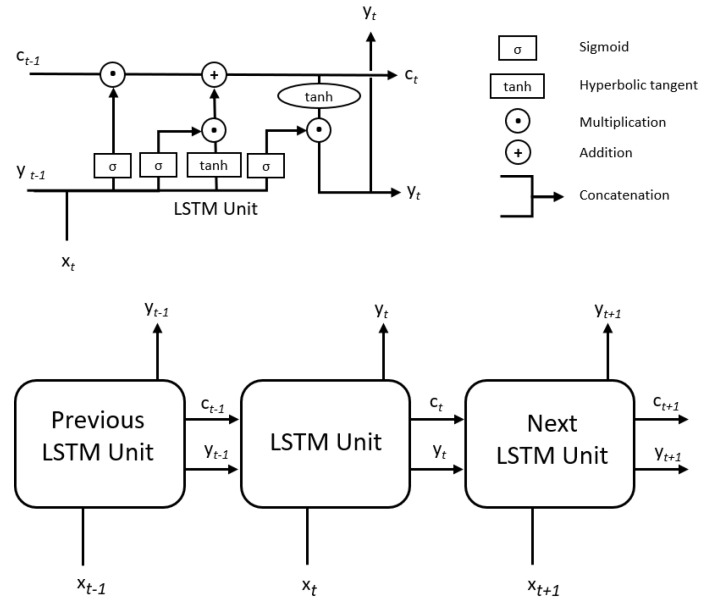
LSTM Univariate. The single input is the value to predict. The output obtained (y${}_{t}$) and the cell state (c${}_{t}$) for each LSTM unit is the input (x${}_{t}$) of the next unit.

To address this problem, the network can remove some of the information that introduces the accumulated error and give more importance to the most recent data such as autoregressive models [26]. Inside an LSTM unit, there is what is known as an internal gate, which is formed by a sigmoid layer and a multiplication operation. Each LSTM memory unit contains three gates that control how the information flows into or out of the unit. The first gate is the input gate which controls when new information can get into the memory. The second gate is known as the forgotten gate which controls when a piece of information is forgotten. This allows less important data to be forgotten, allowing new data to be added. This second gate needs to combine the sigmoid layer with an additional layer formed by a hyperbolic tangent. The last module is the output door which controls that the generated output is saved in the cell state. Each LSTM unit transmits to the next its prediction, which, together with the current input unit, generates the output that is sent as input to the next LSTM unit (see Figure 2).

**Figure 3:**
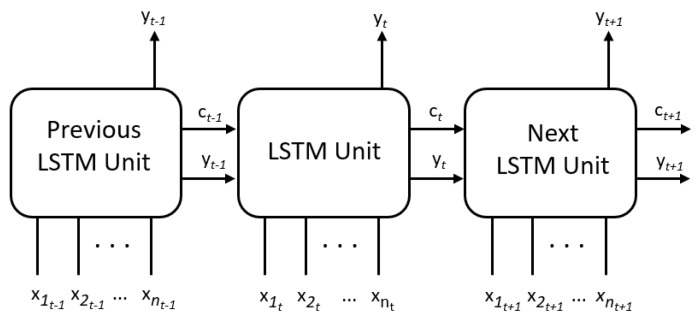
LSTM Multivariate. It is composed of several inputs (*n* represents the number of inputs) and a single output. The first input is the one predicted in the output, as it happens in the univariate model.

In addition to the univariate LSTM scheme, a multivariate LSTM scheme with multiple inputs and a single output is presented (Figure 3). The output used and the input x${}_{1}$ is the same as the univariate LSTM system presented in the previous section. Therefore, the x${}_{1}$ input is the target. This model has the advantage of the additional information provided by the other inputs which implies an increase in the dimensionality of the input layer. This increase in dimensionality makes sense due to the relationship between the variable to be predicted and the rest of the input features added to the multivariate model, which makes the model more robust in its forecast than the univariate model previously presented.

For the multivariate model, several experiments have been performed with different combinations of data, which is explained in detail in Section 3.4.

### 3.3. Description of the Datasets

The validation and training of the proposed LSTM prediction model have been done using the historical data provided by the Sistema de Información Agrario de Murcia (SIAM) (http://siam.imida.es/). This institution has a set of public data that it collects through a set of meteorological stations with different climatic sensors. Since the deployed AIoT system is in Cieza, we have selected the closest weather station to the place of deployment of the weather station to validate and train the proposed model. The objective of validating the proposed model using the public data of this institute is because this institute has meteorological data with a history of years and one of the objectives of this study is to analyze if it is necessary to have a history of data for the proposed LSTM model to obtain more accurate results. In addition, we also want to show that a multivariate model can produce better results than a univariate model. Therefore, the historical dataset requires including, at least, the variables air temperature, humidity and wind speed that the deployed IoT system will have and with which the inference in the intelligent module will be made. The selected SIAM meteorological station is located in the coordinates 38.2839 latitude, −1.49634 longitude, and 244 altitude in the “La Carrichosa” area in Cieza, Región of Murcia, Spain. The measures available in this station are a weather vane, a pluviometer, a radiometer, a thermometer, and a datalogger, the latter to collect all data and avoid losses in case of connection failures. With these meters, the climatic variables (in brackets are the abbreviations used to refer to them) offered by this station are the following:

- Maximum, average, and minimum temperature (TMAX, TMED, TMIN).
- Maximum, average, and minimum relative humidity (HRMAX, HRMED, HRMIN).
- Maximum and average radiation (RADMAX, RADMED).
- Average and maximum wind speed (VVMED, VVMAX).
- Medium wind direction (DVMED).
- Vapor pressure deficit (DPV).

The data provided at that station are collected every hour and this station has had data since 1996; however, for this study, we have started in the year 2012, as in previous years there was a lot of data missing and the model could be affected. The frosts in the area where the present study is focused occur during the month of January and February since this is the period where the temperature changes and the trees are in bloom. Thus, the months selected to assess and validate the model are December, January, and February. Table 1 shows an overview of the datasets, considering the number of instances of each dataset and the input variables to build the LSTM model. It is important to note that the output variable in our LSTM model is always TMIN.

The information contained in each dataset is detailed below:

- DSU-3-month: This dataset contains instances from 1 December 2017 to 28 February 2018, where the input and output variable is the minimum temperature.
- DSM-3-month: This dataset contains instances from 1 December 2017 to 28 February 2018, being composed of the 18 input variables provided by the weather station and the output variable being the minimum temperature.
- DSU-1-month: This dataset contains only January 2018 instances, where the input and output variable is the minimum temperature.
- DSM-1-month: This dataset contains only January 2018 instances, where there are 18 input variables, which come from the weather station and the output variable is the minimum temperature.
- DSUH-1-month: This data set contains only instances from the months of January 2012 to 2018 that have as input and output the variable minimum temperature.
- DSMH-1-month: This data set contains only the instances for the months of January 2012 to 2018. The input variables are the 18 climate variables provided by the weather station for each of the months of January. The output variable is the minimum temperature.
- DSMS-1-month: This last data set contains only the data of January 2018, considering a selection of input variables that influences the minimum temperature, which is actually the output variable.

### 3.4. Experiment Configuration for IoT intelligent Components

This section describes the configuration used for a set of experiments, which aim to assess the quality of the univariate and multivariate LSTM models developed in this work. It is worth highlighting that LSTM models are designed to work with time series data. Thus, in the datasets described in Section 3.3, the data are considered in a temporal way taking samples every hour. Different types of data are considered in each dataset in order to find the best combination of input variables to be considered in the model, as well as time history in data needed that will ensure a minimum error when predicting the minimum temperature. Thus, the anti-frost actuators are only activated when it is convenient, saving water resources. The actuators are configured so that, when the temperature for the next hour is 1.5 Celsius degrees (°C) or lower, these actuators will be activated so that the entire surface can be wetted sufficiently in advance before the temperature falls below zero. A series of experiments will be carried out to evaluate the effectiveness of the method in predicting frost. First, 90% of the data in each set will be used to train the LSTM models and 10% to test and evaluate the model, so that the subset selected for testing has not been used for training and corresponds to the last 10% of the dataset. In other words, the data for the evaluation corresponds to approximately three days for the data sets that only use one month of data. However, when several years of data are considered, it would be evaluating with data corresponding to approximately 10 and 15 days, these days being the last ones of the last month because data considered as time series are being evaluated. Each LSTM model used, both univariate and multivariate, has been optimized by performing a sweep for each of the different parameters indicated below, always considering as the best parameters those whose RMSE is lower, and for the same RMSE value, the value of the parameter that obtains the best R2 has been considered. Table 2 shows the optimized parameters for each LSTM model. Some of these parameters are set in an interval pattern since they depend on the type of experiment. In the evaluation of the quality of the models described, we use the metrics of Root Mean Square Error (RMSE), Mean Absolute Error (MAE), the Pearson Correlation Coefficient (PCC) and Determination coefficient (R2). It is necessary to remark that for all the models we made the task of regression trying to predict the minimum temperature in the following hour to the considered one.

Finally, simulations have been carried out in a GPU-based platform. This platform is composed of Intel(R) Xeon(R) CPU E5-2640 v4 @ 2.40 GHz, 128 GB of RAM, 1 TB SSD Hard Disk, and a NVIDIA GeForce GTX 780 GPU (Kepler). The software environment where the LSTM models has been designed and implemented is based on Tensorflow 1.7.0, Keras 2.1.5, and Python 3.5.2.

## 4. Results and Discussion

This section shows the results obtained for the proposed multivariate LSTM model, comparing its results with the univariate counterpart version. The evaluation data set is described in Table 1. The objective is to find the best set of data and input variables that will provide a model that will be the basis of a decision support system for farmers to reduce their water expenditure, since water is a scarce resource worldwide and even more so in the area where this study is focused.

Table 3 shows the results of the different datasets considering the RMSE, MAE, PCC, and R2 metrics. It is worth highlighting that the main objective is to obtain a model with the least possible error, but, if errors exist, it would be desirable to have them for high temperatures, i.e., above 7 °C. In addition, the output for all datasets is the minimum temperature at the following hour.

Analyzing the results in a general way, the errors of the minimum temperature prediction obtained are not higher than 1.1 °C and the models created have an adjustment higher than 90%. In addition, the difference between the error values of the RMSE and MAE are not high, which indicates that there are no temperature peaks that can be considered outlier and that can be included negatively in the models. However, if the results are deeply analyzed, the DSUH-1-month dataset, obtains the worst error, with an RMSE above 1 °C and a MAE above 0.75 °C. This result is obtained using the univariate LSTM model, where there is only an input variable, i.e., the minimum temperature. The data considered in this input are the minimum hourly temperatures for the months of January 2012 to 2018. This data set assesses whether there is an egalitarian behavior of the data over the years and, using the trend of the previous years, it is possible to predict the values of the following years. As can be seen in the result, although the result is not conceptually bad, it is not as precise as is necessary for the problem posed.

The DSU-3-month dataset is the following configuration according to the results. In this case, the error, although it is less than 1 °C according to RMSE, is still higher than in other configurations. However, the R2 shows that the model is satisfactory, but, from the point of view of precision, it is not as satisfactory as we would like. This dataset uses the same idea as the DSUH-1-month dataset. The main difference in this case is that three months of data history have been considered, taking as input only the minimum temperature and using the univariate LSTM model. The DSM-3-month dataset is the one that continues obtaining a worse result than the previous ones. In this case, the result is very similar to the one obtained by the DSU-3-month dataset that also uses a three-month history. In this case, the DSM-3-month dataset uses the multivariate LSTM model because it takes as input variables all the climatic variables offered by the weather station studied. In this case, the aim is to analyze the place of the temperature trend for each January, if for each hour, using more climatic variables, these influence the prediction of the minimum temperature for the following hour. The RMSE indicates a value of 0.8637 °C and a model fit of 0.9706, but this is still a high error even though the model fit is the best of all settings. The DSMH-1-month dataset has as input variables the 18 climate variables provided by the studied weather station for each hour of January of the years 2012 and 2018. This dataset has an RMSE of 0.8075 °C. Despite being better than the previous ones, it is still a considerable error. The DSU-1-month dataset is the third best model obtained. In this case, the univariate LSTM model is used and as input variable the minimum temperature is taken only from January 2018. The result is very optimistic because it falls below 0.8 °C and the model adjustment achieved is higher than 96%. However, analyzing the results of the datasets mentioned above, there are indications that the multivariate LSTM model obtains a better performance, hence the experiment with the DSM-1-month dataset, where each hour of the month of January is evaluated taking as inputs the 18 climatic variables obtained from the meteorological station studied. With this last multivariate model, an RMSE of almost 0.65 °C is obtained, which improves the previous result. This result has very little room for improvement since it is just over 0.5 °C. However, by analyzing the 18 input variables, a variable selection is made, eliminating those that, according to the literature, may not directly influence the minimum temperature, such as the maximum temperature of the day, the average relative humidity, and the average wind speed. The selection of variables gives rise to the DSMS-1-month dataset, which obtains a lower RMSE and lower MAE, being 0.6353 and 0.5194, respectively, and a satisfactory model fit. This model created using the multivariate LSTM model is the best and therefore is the candidate to build the basis of the decision support system. In addition, this study has given us the classes and needs to extend the proposed AIoT system with more sensors, in particular, with sensors that measure radiation, wind direction, and precipitation.

**Figure 4:**
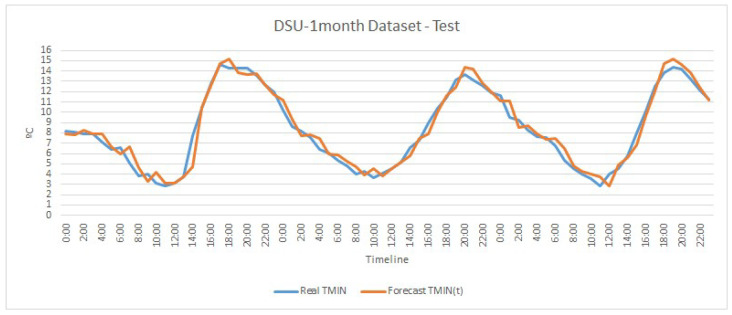
Graphical representation of the actual and predicted minimum temperature values in the test by the univariate LSTM model for the dataset DSU-1-month.

**Figure 5:**
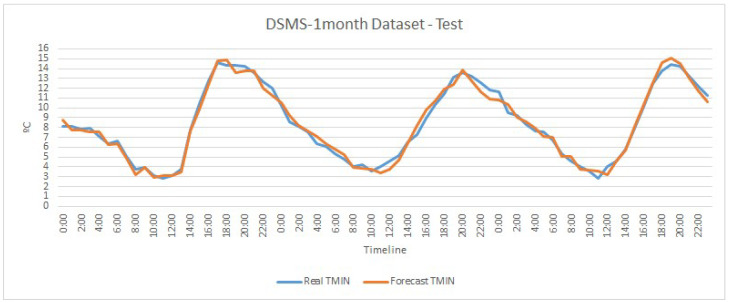
Graphical representation of the actual and predicted minimum temperature values under testing by the multivariate LSTM model for the dataset DSMS-1-month.

Although the differences in the RMSE values are not very large, Figure 4 and Figure 5 depict graphically the trend of the two best models obtained, one for univariate and the other for multivariate, corresponding to the DSU-1-month and DSMS-1-month datasets, respectively.

Figure 4 shows how the model is able to follow temperature trends but has some differences in temperature changes. On the other hand, Figure 5 depicts a greater monitoring of the trend of minimum temperature obtaining a close to perfect prediction in some temperatures. Both models, univariate and multivariate, obtain a greater error in high temperatures, but this does not influence our model, since we seek to be precise in low temperatures to save water and activate anti-frost techniques only when necessary.

**Figure 6:**
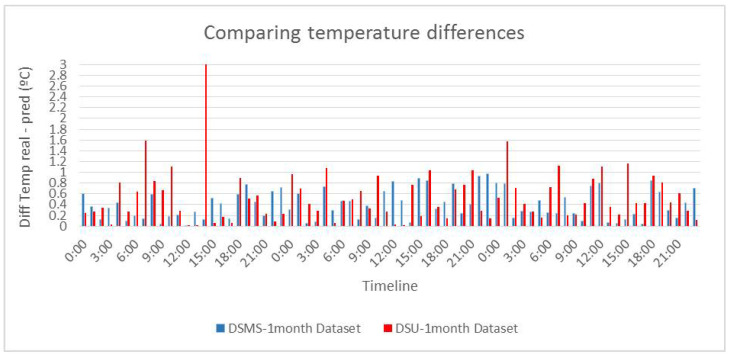
Comparison of the error between the real and the predicted temperature values by the models (under testing) generated with the DSMS-1-month and DSU-1-month datasets.

Figure 6 shows a comparison between the errors, calculated in absolute value, of the actual temperature and the predicted temperature for the datasets DSMS-1month and DSU-1month. This figure allows us to better appreciate the magnitude of errors obtained by univariate and multivariate models. As can be seen, the multivariate model is much more stable in terms of errors and most of them are below 0.7 °C. However, the univariate model is less stable, since although initially it seems to work the same as the multivariate model, it makes errors with a greater variation in temperature, which makes it less robust and stable than the multivariate model.

The results obtained demonstrate the achievement of the objectives initially proposed. These results are satisfactory because they demonstrate a small error and a good adjustment of the models, in comparison with the results obtained in other works presented in Section 2, and our models show lower RMSE and higher $R^{2}$ ratios.

## 5. Conclusions and Future Work

Climate change is causing trees to flower earlier and sudden changes in temperature can cause serious economic damage to farmers by freezing flowers and damaging crops. In addition, there is often unnecessary water wastage in areas particularly vulnerable to drought. This article proposes an intelligent IoT system to optimize water use in crops by accurately predicting minimum air temperature at plot level and thus limiting the use of the anti-frost sprinkler irrigation techniques. The intelligent component in the IoT infrastructure proposed is the key point for decision-making. It is designed using an approach based on a multivariate LSTM model which accurately provides air temperature predictions with enough time in advance to take actions to avoid losing the crop. The best configuration of the proposed LSTM multivariate model will be integrated into the IoT system as a decision support component. The objective of the proposed model is to achieve the least possible error so that the farmer can trust the system and the system only activates the irrigation sprinklers to avoid frost when the temperature will be less than or equal to 1.5 °C, this activation being reliable based on the prediction of the temperature in the following hour. In the result analysis of the proposed multivariate LSTM model, we have done comparisons with the univariate LSTM model using different combinations of input variables that can influence the temperature prediction. The best results obtained are satisfactory as they indicate that the model that obtains a lower RMSE is the LSTM multivariate model that uses only data from the same month to train and nine climatic input variables to predict the minimum air temperature. Thus, the results of the proposed multivariate LSTM model obtain an RMSE of 0.64° and a fit value of the R2 model of 0.97. In the future, we will work on analyzing the possibility of implementing the multivariate model at the edge of the IoT infrastructure to prevent transient cloud outages and improve performance. Other intelligent services will be provided such as the prediction of evaporation in rafts to optimize agriculture procedures and other different applications will be deployed such as the climate control in greenhouses.

## Figures and Tables

**Table 1:** Description of the datasets used for model validation, indicating number of instances of each dataset (N.Instances) and input variables (Input variables). The acronyms of these variables mean: TMED- mean temperature, TMAX- maximum temperature, TMIN-minimum temperature, HRMED-mean relative humidity, HRMAX-maximum relative humidity, HRMIN- minimum relative humidity, RADMED-mean radiation, RADMAX- maximum radiation, RADACU- accumulated radiation, VVMED-mean wind speed, DVMED- mean wind direction, VVMAX-maximum wind speed, PREC-rainfall, DEWPT- dew point, and DPV-pressure vapor deficit.

Dataset	N.Instances	Input Variables
DSU-3-month	2160	TMIN
DSM-3-month	2160	TMED, TMAX, TMIN, HRMED, HRMAX, RMIN, RADMED, RADMAX, RADACU, VVMED, DVMED, VVMAX, PREC, DEWPT, DPV
DSU-1-month	744	TMIN
DSM-1-month	744	TMED, TMAX, TMIN, HRMED, HRMAX, RMIN, RADMED, RADMAX, RADACU, VVMED, DVMED, VVMAX, PREC, DEWPT, DPV
DSUH-1-month	4464	TMIN
DSMH-1-month	4464	TMED, TMAX, TMIN, HRMED, HRMAX, HRMIN, RADMED, RADMAX, RADACU, VVMED, DVMED, VVMAX, PREC, DEWPT, DPV
DSMS-1-month	744	TMIN, HRMAX, RADMAX, RADACU, VVMED, DVMED, PREC, DEWPT, DPV

**Table 2:** Optimal parameter for LSTM execution experiments for the univariate and multivariate models.

Parameter	Univariate Model Values	Multivariate Model Values
Number of input neurons	70	60:90
Batch size	32	32
Number of epochs	230	340:750
Learning factor	0.001	0.001
Optimizer	Adam	Adam
Activation function	Hyperbolic Tangent	Hyperbolic Tangent
Loss Function	Quadratic Mean Error	Quadratic Mean Error
Delay Sequence	6	6

**Table 3:** Results obtained applying univariate and multivariate LSTM models to the datasets according to the different datasets and configurations of the input variables. For each dataset, the Root Mean Square Error (RMSE), the Mean Absolute Error (MAE), the Pearson Correlation Coefficient (PCC), and the determination coefficient (R${}^{2}$) are provided. Results ordered from highest to lowest RMSE.

Dataset	RMSE (${}^{{}^{\circ}}$C)	MAE (${}^{{}^{\circ}}$C)	PCC	R${}^{2}$	LSTM Model
DSUH-1-month	1.0641	0.7636	0.9717	0.9442	Univariate
DSU-3-month	0.8860	0.6172	0.9844	0.9691	Univariate
DSM-3-month	0.8637	0.6842	0.9854	0.9706	Multivariate
DSMH-1-month	0.8075	0.6223	0.9761	0.9517	Multivariate
DSU-1-month	0.7227	0.5451	0.9814	0.9613	Univariate
DSM-1-month	0.6495	0.5309	0.9853	0.9687	Multivariate
DSMS-1-month	0.6353	0.5194	0.9856	0.9701	Multivariate
